# Inflammation and Heart Failure: Searching for the Enemy—Reaching the Entelechy

**DOI:** 10.3390/jcdd10010019

**Published:** 2023-01-04

**Authors:** Ioannis Paraskevaidis, Dimitrios Farmakis, Georgios Papingiotis, Elias Tsougos

**Affiliations:** 1 Medical School, National and Kapodistrian University of Athens, 11527 Athens, Greece; 2 6th Department of Cardiology, Hygeia Hospital, 15123 Athens, Greece; 3 Medical School, University of Cyprus, 2029 Nicosia, Cyprus; 4 Department of Cardiology, Attikon University Hospital, National and Kapodistrian University of Athens, 12462 Athens, Greece

**Keywords:** heart failure, inflammation, pathophysiology, homeostasis, inflammasome, autoimmunity

## Abstract

The pivotal role of inflammation in the pathophysiology of heart-failure (HF) development and progression has long been recognized. High blood levels of pro-inflammatory and inflammatory markers are present and associated with adverse outcomes in patients with HF. In addition, there seems to be an interrelation between inflammation and neurohormonal activation, the cornerstone of HF pathophysiology and management. However, clinical trials involving anti-inflammatory agents have shown inconclusive or even contradictory results in improving HF outcomes. In the present review, we try to shed some light on the reciprocal relationship between inflammation and HF in an attempt to identify the central regulating factors, such as inflammatory cells and soluble mediators and the related inflammatory pathways as potential therapeutic targets.

## 1. Introduction

Heart failure is a common syndrome in western communities and despite the advances of the last decades, the rates of morbidity and mortality remain high [1]. Although the pathophysiology of this entity has been thoroughly investigated, many questions remain unanswered. In addition to the activation of the sympathetic nervous system and the renin-angiotensin-aldosterone system, which are considered the cornerstones of the syndrome’s pathophysiology and management, the role of inflammation has been widely discussed for many years. There further seems to be an interrelation between neurohormonal activation on one hand and inflammation and free radical production on the other. Indeed, many scientific reports suggest the reciprocal relationship of heart-failure syndrome and inflammation. This has been documented by the presence of high blood levels of pro-inflammatory and inflammatory indexes and their association with adverse outcomes in patients with heart failure [2,3,4]. Thus, the pivotal role of inflammation in the pathophysiology of heart-failure development and progression is well recognized. Moreover, there is solid evidence supporting the hypothesis that inflammation and redox disorders are linked with arrhythmia burden [5]. However, anti-inflammatory therapeutic modalities have not yet had a significant impact in cardiovascular medicine since the results of the clinical studies have been ambiguous. The Canakinumab Anti-inflammatory Thrombosis Outcome Study (CANTOS) and the Colchicine Cardiovascular Outcomes Trial (COLCOT) were the first two randomized clinical trials that showed promising results in the prevention of cardiovascular medicine [6,7]. However, there are several things to be addressed in the direction of dealing with inflammation in cardiovascular health and the clinical implementation of anti-inflammatory therapies. It seems that early initiation of anti-inflammation therapy has a beneficial effect on the heart [8]. Have we really understood the nature of this reciprocity? Is it possible that there is a central mediator, a “master key” that regulates both conditions and is not yet well defined? It would therefore be of interest to attempt a search for this ‘master key’ that might regulate the relationship between inflammation and heart failure, further providing a potential therapeutic target.

## 2. Inflammation

**Figure 1:**
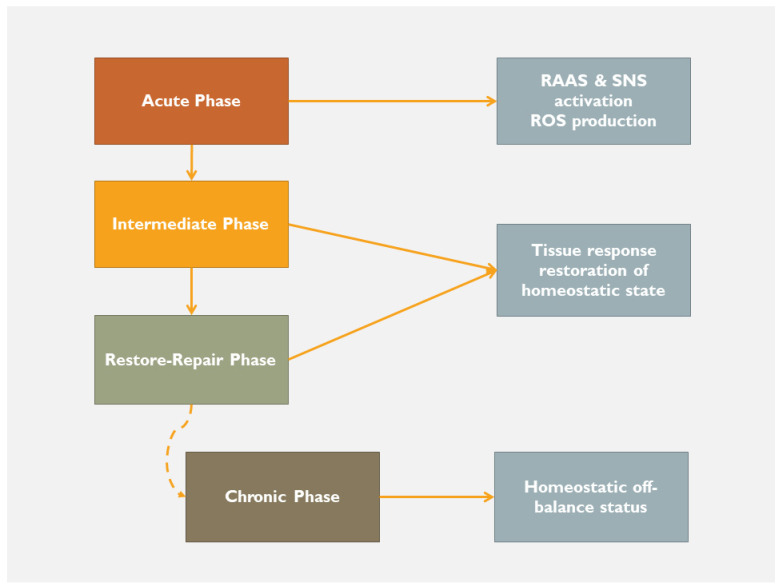
Myocardial inflammatory response to an insult (RAAS—renin-angiotensin-aldosterone system; SNS—sympathetic nervous system; ROS—reactive oxygen species).

Inflammation is defined as the response of the immune system to a variety of stimuli that might be infectious or tissue harmful. Regardless of the initial insult, there is a series of programmed sequelae depending on the ability of the immune system to eliminate the ‘enemy’ and restore the tissues’ normal structure and function. The inflammatory process can be divided, without clearly defined and therefore overlapping borders, into three sequential phases, including the acute phase, the intermediate and the restore/repair phase. However, an alteration of this physiological sequence may occur, leading potentially to a different homeostatic status, namely a chronic phase of inflammation, which might evolve into a catastrophic pathway (Figure 1).

**Figure 2:**
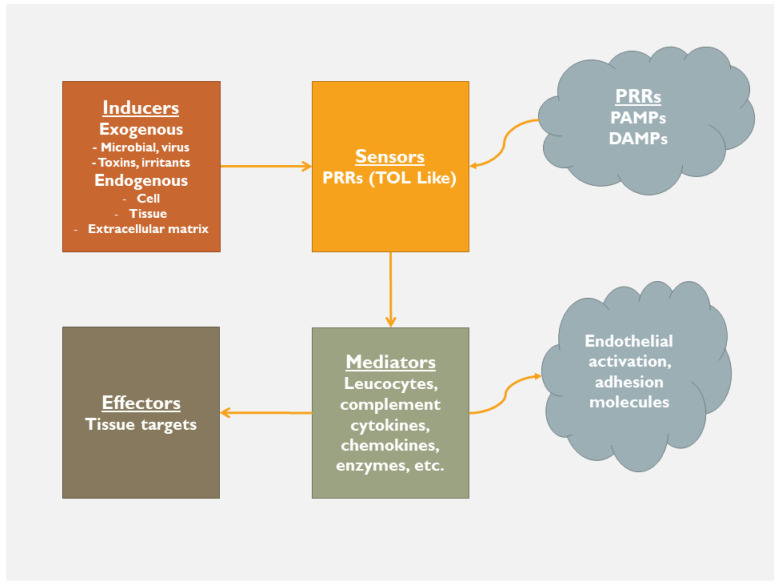
Mechanisms and mediators involved in the inflammatory process (PRRs—pattern recognition receptors; PAMPs—pathogen associated molecular patterns; DAMPs—damage-associated molecular patterns).

**Figure 3:**
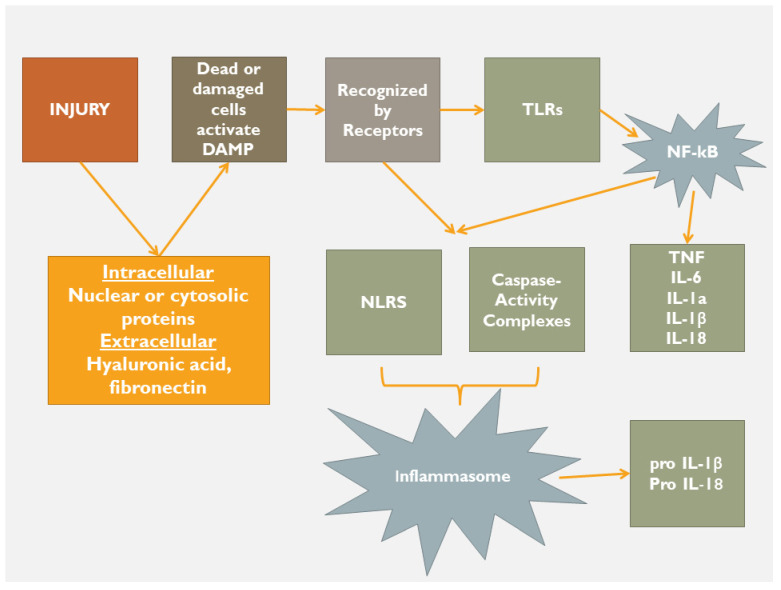
Mechanisms and mediators involved in sterile inflammation (DAMP—damage-associated molecular patterns, TLRs—Toll-like receptors; NF-kB—nuclear factor kappa-beta; NLRs—NOD-like receptors; IL—interleukin).

In the early phase, there is a stepwise process that includes inducers (exogenous, endogenous), sensors (pattern recognition receptors), mediators (leucocytes, cytokines etc.) and effectors (tissue targets; Figure 2 and Figure 3). In this early phase of inflammation, there is an activation of the bone marrow and splenic cells, along with the activation of circulating blood cells (leucocytes, mast cells, dendritic cells, etc.) [9]. At the same time, T-naïve lymphocytes are called to produce different pre-inflammatory substances that might be either protective [e.g., interleukin (IL)-10] or harmful (e.g., IL-6, 18, 1b) [9]. The target of this first phase is to eliminate necrotic tissue by activating protection-elimination mechanisms such as autophagy, mitophagy, degradation, fragmentation, etc., aiming at structural restoration and repair. Along with the structural restoration/repair effort, there is neurohormonal activation (renin-angiotensin-aldosterone system, sympathetic nervous system, natriuretic peptide system) that is involved in order to maintain cardiac output, tissue perfusion and oxygenation and hence to support the basic metabolic needs of peripheral tissues.

The end of this early phase signals the beginning of the intermediate and the ensuing repair phase, the latest identified by the up-coming interaction between multicellular protein expression, specialized matrix-protein activation (fibronectin, osteopontin, proteoglycans, etc.) along with cellular sources of participation (cardiomyocytes, fibroblasts, macrophages, vascular cells, structural extracellular matrix), thus leading to the regulation of inflammation and healing response [10]. To do so, the homeostatic process has to proceed to the final maturation phase, in order to restore cardiac function and to satisfy the metabolic needs of peripheral tissues. In the case of failed or deviated maturation, heart failure emerges (Figure 1 and Figure 3) [11]. At this very crucial point, there may be a down-regulation of lipid mediators (lipoxins, resolvins, protectins, etc.) [12,13] and an over-activation of toll-like receptors [14], leading to a new homeostatic status, signaling the chronicity of the homeostatic distortion [15,16,17], thus accentuating cardiac adverse remodeling. As far as this is true, the emerging question, from this point and beyond, is whether the initial index event or the subsequent homeostatic imbalance produces the condition that might be named the real ‘enemy’. Do we face the birth of a self-destruction mechanism? Has the homeostatic process reached its entelechy? There is a need for further investigation in these potential disease-relevant pathways of inflammation and homeostasis. Indeed, if the body fails to eliminate the enemy, new characteristics of inflammation emerge, signaling a new homeostatic status. A status that is dynamic, involves many feedback systems and adapts to the internal environment. Thus, we face a step forward of the homeostasis that passes from the acute face to a different type of adaptation indicating chronic inflammation (Figure 1) [18]. Several reports suggest that passing from the acute to chronic phase is a key step, beyond which heart failure manifests [19,20,21,22,23].

### 2.1. Following a Self-Catastrophic Path—Missing the Balance

**Figure 4:**
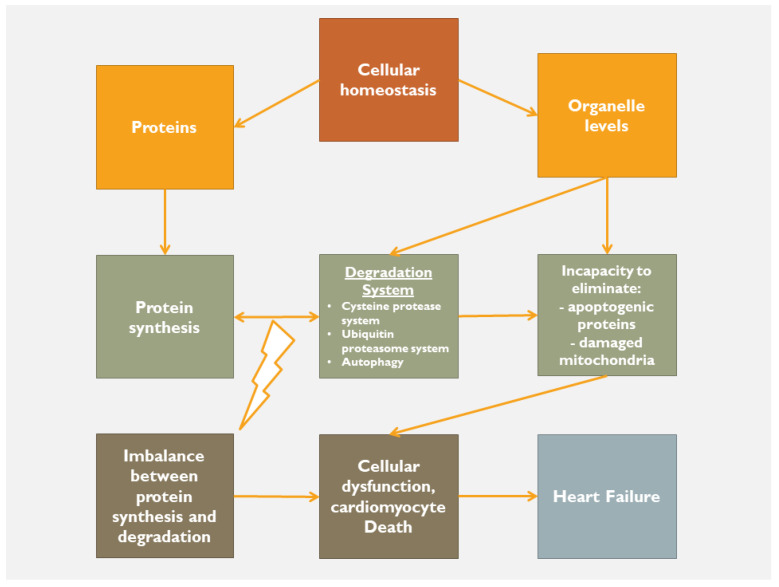
Deranged homeostasis leading to heart failure.

Following an acute index event, the body, as a whole, tries to retain its homeostatic status. If the cause is of minimal aggressiveness, then the homeostatic status remains within normality by using low adaptation mechanisms. However, in the case of a major index event, the body tries to maintain homeostatic status by any means in order to limit the cause, to heal, resolve and ultimately to repair the tissues’ structure and function. In this respect, when a severe disturbance of homeostasis occurs, then the inflammatory process is activated as the acute-intermediaterestore phase, followed, in case of failure of the above-described sequence, by the chronic phase. Regardless of the cause of a sterile inflammation, there is tissue damage and consequently a release of intracellular (nuclear and/or cytosolic proteins, etc.) and extracellular (hyaluronic acid, fibronectin, etc.) products (Figure 3). The release of these proteins activates a series of injury-associated molecular pathways through cardiac receptor signaling. At the beginning, release of inflammatory cytokines, neutrophil aggregation and activation, release of proteases and ROS production occur. Failure of this initial reaction to restore tissue integrity activates a forward step of inflammation, in which the toll and nucleotide binding and oligomerization domain (NOD)-like receptors (NLRs) are involved with further accumulation and activation of pro-inflammatory mediators. At this crucial phase, it is very important to maintain equilibrium between protein degradation (cysteine-protease system, ubiquitin proteasome, autophagy, etc.) and protein synthesis. If this equilibrium fails, apoptogenic mediators, misfolded proteins and damaged mitochondria lead to the phase of chronic inflammation (Figure 4). The NLRs, joined by caspase-activity complexes, form the inflammasome (Figure 3) that further stimulates the production of IL-1b and IL-18 that affect left ventricular systolic function, alter mitochondrial function and decrease sympathetic activity [24,25].

The role of NLRP3 inflammasome (NLR family, pyrin domain-containing 3) in heart failure is well documented [26,27,28]. NLRP3 inflammasome sets off the maturation of proinflammatory cytokines (IL-1β and IL-18) to initiate the inflammatory response and plays a key role in modulating chronic inflammation, altering the physiological adaptation of cardiomyocyte and leading to heart failure progression [26]. Recent data showed that two other inflammasomes seem to be involved in the inflammatory process in failing hearts. Inflammasome protein absent in melanoma 2 (AIM2) and NLR family CARD domain-containing protein 4 (NLRC4) have been found to be over-expressed and activated in human-heart tissues as well in vivo animal models. These two other inflammasomes may contribute to the chronic inflammation in heart failure and also a therapeutic target [27]. The inflammasome also defines the interplay between innate and adaptive responses, paving the way toward the development of heart failure. Furthermore, the involvement of the immune process (effect of T and B cells) promotes chronicity according to the self-antigen hypothesis, the production of autoantibodies and tissue fibrosis, suggesting a role for autoimmune mechanisms [22,29]. This self-protection/elimination process integrates the endogenous inducers, cell-, tissue-, plasma- and extracellular matrixderived signals and might develop in an uncontrolled manner. Any injured myocardial cells can maintain a basal, stressed, apoptotic or necrotic state. If the amount of injured tissue is enormous and overpasses the homeostatic capacity to restore cell-tissue normality, then the detrimental chronic inflammatory phase develops [30]. On the other hand, the successful restoration of homeostasis prevents the harmful effect of chronic inflammation [31,32].

### 2.2. Homeostatic Mechanisms

To achieve homeostasis, a balanced activity between protein synthesis-degradation and organelle capacity to eliminate apoptogenic proteins and damaged mitochondria should be activated and well-functioning. If this is not the case, then the cardiomyocyte death along with extra-cellular cardiac matrix dysregulation, lead to myocardial cellular dysfunction and ultimately to heart failure (Figure 4). In other words, the body tries to protect itself from itself. Indeed, when mitochondrial morphology and function are disturbed (lack of fission, fusion and hence mitophagy), mitochondrial DNA is released into cytosol, and along with the misfolded proteins and the activation of the mitochondria-associated endoplasmic reticulum membranes (MAMs), promotes the enhancement of a self-destruction process, that might involve the entire body [33,34]. In case of a cardiac harmful event, there is an activation of danger-associated molecular patterns (DAMP) released by the nucleus (e.g., DNA, RNA), the mitochondria (e.g., DNA) and the cytosol (e.g., RNA). In this respect, regardless of the initial triggering event (pressure overload, volume overload, myocardial infarction, etc.), there is an activation of an inflammatory process associated with the harmful release of cell proteins along with the activation of the aforementioned self-elimination/protection mechanism. Thus, if there is an imbalance of this sequel, then the chronic inflammation is switched on, and in case of an uncontrolled process, heart failure develops. In other words, it seems that if the homeostatic mechanism (degradation system, autophagy, etc.) is successful, inflammation is limited. On the other hand, if the homeostatic protective mechanism cannot control and limit the harmful events, the self-catastrophic pathway promotes cardiomyocyte death and hence heart failure. The inevitable question that arises is whether the cause of heart failure is inflammation per se or the incapacity of the homeostatic protective mechanisms.

Damaged and un-repaired mitochondria are the source of reactive oxygen species, and along with mitochondrial DNA release, generate proinflammatory cytokines and the activation of inflammasome, promoting inflammation chronicity. This leads to an increase of the rate and amount of myocardial cell death and hence to the development of heart failure. Although the role of inflammasome (and its subfamilies) is not very well understood, it appears that its formation and activation have dual contradictory roles. The first one is to eliminate the ‘enemy’ and restore the normal anatomy and function of the tissue, while the second one, under certain circumstances, could be harmful by distorting the normal activity, which is to avoid chronic inflammation and to promote the protective mechanisms of homeostasis; in other words, to recognize the released material as foreign and to attack these unrecognized substances in order to ‘protect’ the cell and consequently the normal anatomy and function of the tissue [35,36].

It should be stressed that cardiomyocyte homeostasis as described above is different from heart (organ) and body homeostasis. The heart as an organ tries to adapt to stressors and noxious agents mediated by inflammation and redox disorders with an effort to maintain its function in the human body.

### 2.3. Organelle Communication

The normal function of a cell depends mainly on the structural functional integrity of its constituents, the organelles. The endoplasmic reticulum (ER) is an organelle that regulates important intracellular function, including protein synthesis, calcium transportation, etc. In the case of an index event, the ER is stressed and tries to maintain normality through homeostasis. In fact, ER-associated degradation, the unfolded protein response, reticulophagy, proteostasis, autophagy, etc., are activated in order to maintain normality [37,38,39]. In addition, there is communication with the other organelles, lysosomes, mitochondria, plasma membrane, etc., thus facilitating the normal functions of the cell, including lipid metabolism [40], calcium homeostasis [37,41], ion exchange [40], etc. However, if the index event surpasses the capacity of the cell to retain homeostasis or if ER homeostatic properties are impaired, then the cell-defending mechanisms fail, thus leading to a possible harmful path [42,43,44].

Although there is vast communication among the organelles, it seems that the most important one is between the ER and mitochondria [45,46]. Indeed, these two organelles form the ER-mitochondria contacts (ERMCs) [47], constituted by both lipid and protein complexes [48]. Studies have demonstrated that ERMCs are involved in the progression of several cardiovascular diseases [40,49,50,51,52,53], because they are involved in several biological processes, such as calcium homeostasis, apoptosis, autophagy, protein synthesis and folding, inflammation etc. [54,55,56,57,58,59,60,61]. After an index event, misfolded proteins are accumulated in the ER promoting the activation of the unfolded protein response in order to maintain proteostasis. In the case of failure of the misfolded protein repair, or of a large amount of accumulated unfolded proteins, a vicious circle begins [62,63]. This vicious circle is characterized by the loss of homeostatic capacity, promoting apoptosis. However, ER activation facilitates steroid synthesis, ER stress, phospholipid metabolism in mitochondria, autophagy and apoptosis [63], and under certain circumstances can increase transcription-factor expression (ATF) 6 and 4 and promote apoptosis either alone or in cooperation with mitochondria [64,65,66]. A self-catastrophic sequence thus begins. Indeed, when the collaboration between these two organelles is impaired, a progression to advanced heart failure may occur [67,68]. In fact, it has been stated that uncontrolled ER stress provokes distortion of myocardial architecture, alteration of mitochondrial metabolism and function, leading to an energy deficiency, along with a reduction of calcium transfer and consequently impairment of cardiac contractility and relaxation, hence heart failure [69,70].

### 2.4. Targeting Inflammation, Oxidative Stress and Mitochondrial Dysfunction

Regardless of whether the inflammation is the cause or the consequence of heart failure, it remains an important factor and a potential therapeutic target [71]. Although, several studies have been conducted in order to investigate the role of anti-inflammatory therapies, the results have hitherto been poor or controversial [72]. Notably, anti-cytokine therapies were tested in the ATTACH and RENEWAL studies with poor results [73,74]. On the other hand, the CANTOS trial has shown that the inhibition of IL-1b with canakinumab was followed by a significant trend for a dose-dependent reduction in the incidence of the composite endpoint of hospitalization for heart failure and heart failure-related mortality [75]. However, this was not the case in other studies, showing that after IL-1b inhibition with canakinumab, substantial residual inflammatory risk remained, related to both IL-18 and IL-6 [76]. Other studies based on anti-inflammatory therapies have been published [77,78,79], among which those using either immunomodulation [80] or anti-inflammatory drugs [81,82,83], showing overall poor results. The same was true when N-terminal pro-B-type natriuretic peptide (NT-pro BNP) or high-sensitivity C-reactive protein (hs-CRP) were used as endpoints [84,85].

These data support the need for a better understanding of the inflammatory process. As it has been pointed out, important inflammatory mediators are released after the activation of the inflammasome, suggesting that the inflammasome could be a therapeutic target. Since the inflammasome is part of homeostatic mechanism, one could speculate that homeostatic controlled response is the master key to investigate and target.

Regarding oxidative stress, its role in pathogenesis of heart disease and heart failure has been thoroughly studied [86,87]. The clinical studies examining the effects of several anti-oxidative strategies have not shown the beneficial effects that preclinical studies described [87]; however, innovative antioxidant perspectives are worth being evaluated. Targeting glutamyl cycle or NAD^+^ production, the endogenous antioxidant capacity of the cardiomyocyte may be of interest in targeting new treatment modalities in heart-failure patients [88].

As far as mitochondrial dysfunction is concerned as an approach for therapy to improve cardiac function directly, several pathways have been marked as potential pharmacologic targets, such as blocking increased reactive oxygen species, blocking mitochondrial permeability transition pores (MPTP), improving the efficiency of electron-transport complexes and regulation of mitochondrial ion homeostasis [89]. Different molecules (mitoquinone, elamipretide, CGP-37157, cariporide, etc.) have been proposed as therapeutic agents targeting each of the possible above-mentioned pathways; however, further research is warranted in bioenergetic insufficiency in heart failure [90].

Leaving apart all these pharmacological targets, we have to consider that in patients with heart failure, exercise-based approaches have been shown to improve quality of life and functional capacity and to reduce hospital admissions [1]. The pivotal anti-inflammatory role of exercise training has been suggested to be a large number of mediators including macrophages [91,92,93,94,95,96,97]. In contrast to pharmacological interventions, exercise training ameliorates the inflammatory profile, suggesting the capacity of the body to restore the deviation. Is this because we pharmacologically target the inflammasome products rather than the inflammasome per se? In addition to exercise, neuromodulation with low-level transcutaneous vagus nerve stimulation in a pilot randomized clinical trial showed an improvement in cardiac function and in inflammatory cytokines profile in patients with heart failure with preserved ejection fraction [98].

Several studies have shown no promising results even when they used NLRP3 inhibitors [99,100,101,102,103,104,105] or caspase-1 inhibition [106,107,108,109,110,111]. Furthermore, other studies using inhibition of other subunits of the inflammasomes NLRP 1, 6-7, 12, NAIP, NLRC4, and AIM2 show their unknown role in the inflammation process [112,113,114,115,116,117,118,119,120,121,122,123,124,125,126,127,128,129].

Do we have to suppress inflammasome activity? Is the inflammasome the corner stone of the process? How can we suggest depressing the first defensive mechanism? Shortly, what must be the therapeutic goal—o target the mediators or the inflammasome products? Or do we have to somehow find the way and the tools to re-organize the normal homeostatic status?

Data from the A systems BIOlogy Study to TAilored Treatment in Chronic Heart Failure (BIOSTAT-CHF) study cohort have introduced some potential therapeutic targets, such as the blockage of inducible costimulator ligand (ICOSLG), TNF superfamily member-14 (TNFSF14), CD28, CD70 and the enhancement of interferon-γ production [130].

## 3. Conclusions

Inflammation in heart failure is a very complex process and many factors, some of them totally unknown, are involved. It seems that on the way to finding out the interaction between inflammation and heart failure, we might miss the real ‘enemy’ that is the deranged and malfunctioning homeostatic properties. A better understanding of inflammatory pathways in cardiomyocyte damage would allow for potential therapeutic targets, pharmacological and non-pharmacological. The research continues; the questions have been set out and the long way towards entelechy has begun.

## Data Availability

Not applicable.
